# Author Correction: Emerging photoluminescence from the dark-exciton phonon replica in monolayer WSe_2_

**DOI:** 10.1038/s41467-019-12595-7

**Published:** 2019-10-11

**Authors:** Zhipeng Li, Tianmeng Wang, Chenhao Jin, Zhengguang Lu, Zhen Lian, Yuze Meng, Mark Blei, Shiyuan Gao, Takashi Taniguchi, Kenji Watanabe, Tianhui Ren, Sefaattin Tongay, Li Yang, Dmitry Smirnov, Ting Cao, Su-Fei Shi

**Affiliations:** 1 Department of Chemical and Biological Engineering, Rensselaer Polytechnic Institute, Troy, NY 12180 USA; 2 School of Chemistry and Chemical Engineering, Shanghai Jiao Tong University, 200240 Shanghai, China; 3 Kavli Institute at Cornell for Nanoscale Science, Ithaca, NY 14853 USA; 4 National High Magnetic Field Lab, Tallahassee, FL 32310 USA; 5 Department of Physics, Florida State University, Tallahassee, FL 32306 USA; 6 College of Physics, Nanjing University, 210093 Nanjing, China; 7 School for Engineering of Matter, Transport and Energy, Arizona State University, Tempe, AZ 85287 USA; 8 Department of Physics, Washington University in St. Louis, St. Louis, MO 63136 USA; 9 National Institute for Materials Science, 1-1 Namiki, Tsukuba, 305-0044 Japan; 10 Geballe Laboratory for Advanced Materials, Stanford University, Stanford, CA 94305 USA; 11 Department of Materials Science and Engineering, University of Washington, Seattle, WA 98195 USA; 12 Department of Electrical, Computer & Systems Engineering, Rensselaer Polytechnic Institute, Troy, NY 12180 USA

**Keywords:** Two-dimensional materials, Fluorescence spectroscopy

Correction to: *Nature Communications* 10.1038/s41467-019-10477-6, published online 06 June 2019.

The original version of this Article omitted the following from the end of the Acknowledgements: ‘Z.Lu and D.S. acknowledge support from the US Department of Energy (DE-FG02-07ER46451) for magneto-optical measurements performed at the National High Magnetic Field Laboratory, which is supported by the National Science Foundation Cooperative Agreement No. DMR-1644779 and the State of Florida’. This has now been corrected in both the PDF and HTML versions of the Article.

