# Supplementary material for: Emerging photoluminescence from the dark-exciton phonon replica in monolayer WSe2
Source: Nat Commun. 2019 Jun 6;10:2469. doi: 10.1038/s41467-019-10477-6 (PMC6554274; doi:10.1038/s41467-019-10477-6)
Supplement: Supplementary file 1 — Supplementary Information [file 41467_2019_10477_MOESM1_ESM.docx]

**Emerging Photoluminescence from the Dark-Exciton Phonon Replica in Monolayer WSe_2_**

Zhipeng Li et al.

**Supplementary Note 1. Device fabrication**

The BN encapsulated monolayer WSe_2_ device was fabricated by the PPC (polypropylene carbonate) pickup method following the previous work^1,2^. Here we also describe the process briefly as following: first, the monolayer WSe_2_, few-layer graphene, and few-layer BN were exfoliated onto Si wafer with 285 nm thermal oxide and inspected under the optical microscope. A microscope slide with ~ 1 μm thick PPC on the transparent elastomer stamp (PDMS, poly dimethyl siloxane) was inverted and attached to the transfer stage. We picked up the few-layer BN flake by the PPC film. The attached BN was used as the stamp to pick up the monolayer WSe_2_, few-layer graphene, and another few-layer BN flake sequentially. The prepared stack was placed onto the pre-patterned Au electrodes. The PPC was removed by heating the stack up to 90 °C. We then used chloroform to remove the PPC residue, and the resulted BN/WSe_2_/Graphene/BN stack was left on the substrate. Finally, we added another few-layer graphene flake onto the top BN to work as a top gate electrode, using the top BN as the dielectric.

**Supplementary Note 2. Magneto-optical measurement setup**

The magneto-PL setup is shown in Supplementary Fig. 1. The incident laser goes through a linear polarizer and a beam splitter, then the linear polarized light is converted into circularly polarized light by the λ/4 waveplate and the laser is focused by a 32X objective (NA: ~ 0.6) to a spot size of ~ 2 μm to excite the WSe_2_ sample. The PL is collected with the same objective and goes through the quarter waveplate to be converted into linear light. The linear light passes the beam splitter, the λ/2 waveplate and the second linear polarizer, and is detected by the CCD camera attached to the spectrometer. The assembly of the λ/2 waveplate and the second linear polarizer is used to distinguish the ($\sigma^{+},\sigma^{+}$) and ($\sigma^{-},\sigma^{-}$) configurations.

**
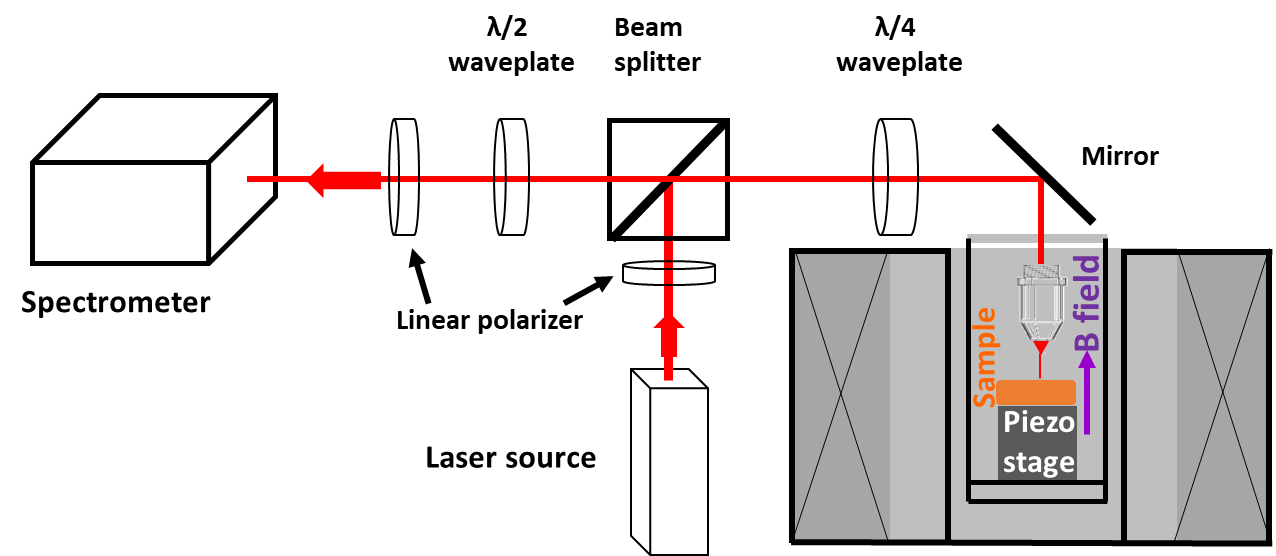
**

**Supplementary Figure 1. Schematic of the magneto-optical setup for the PL measurement**

**Supplementary Note 3.** $\boldsymbol{g}$**-factor calculation**

Based on the tight binding model^3–5^, orbital contribution $g_{O}$, valley contribution $g_{valley}$, and spin contribution $g_{S}$ are the three contributing components in the general formula for the *g*-factor of each band.

The Zeeman energy split is thus denoted by the following equations:

$E_{C,V}^{K(K^{'})}=E_{C,V}^{0}+\frac{1}{2}(\tau_{z}g_{valley}+\tau_{z}g_{O}+\sigma_{z}g_{S}) \mu_{B}B$ (1)

$E^{K(K^{'})}=E_{C}^{K(K^{'})}{-E}_{V}^{K(K^{'})}- E^{b}$ (2)

$\Delta E=E^{K}{-E}^{K^{'}}$ (3)

$E_{C,V}^{K(K^{'})}$ corresponds to the position of the Zeeman shifted conduction band minimum (CBM) or valence band maximum (VBM) for K ($K^{'}$) valley. $E_{C,V}^{0}$ is the energy of the CBM or VBM at the absence of the magnetic field. $E^{K(K^{'})}$ is the the Zeeman shifted PL peak position of each excitonic complexe for the K or $K^{'}$ valley. $E^{b}$ is the binding energy for the particular excitonic complex interested. $\Delta E$is the energy difference between $E^{K}$ and $E^{K^{'}}$. With a simplified model^6,7^ in which only d-orbital components are considered in both the valence band minimum (VBM) and conduction band maximum (CBM) of monolayer WSe_2_, the detailed contributions to the *g*-factor for different parameters are shown in Supplementary Table 1.

**Supplementary Table 1. The values for different parameters**

| $\boldsymbol{\sigma}_{\boldsymbol{z}}$ | $\boldsymbol{\tau}_{\boldsymbol{z}}$ |  | $\boldsymbol{g}_{\boldsymbol{O}}$ | $\boldsymbol{g}_{\boldsymbol{valley}}$ | $\boldsymbol{g}_{\boldsymbol{S}}$ |
| --- | --- | --- | --- | --- | --- |
| +1 for spin up | +1 for K | CB | 0 | $2 (m_{0}/me )$ | 2 |
| -1 for spin down | -1 for K’ | VB | 4 | $2 (m_{0}/mh )$ | 2 |

Where $m_{0}$ is the mass of the free electron, $m_{h}$ is the effective mass of the hole and $m_{e}$ is the effective mass of the electron.

As a result, the dark exciton should have the spectral *g*-factor:$g=-8+2{{{(m_{0}}/{m_{e}-}m}_{0}}/{m_{h}})$, which is -8 if assuming electron-hole symmetry, $m_{e}=m_{h}$. The calculated *g*-factors for exciton, dark exciton and trion-exciton complexes are shown in Supplementary Table 2.

**Supplementary Table 2. The calculated *g*-factors for** **the exciton, dark exciton and trion-exciton complex.**

| Peaks | Spectral *g*-factor |
| --- | --- |
| Bright exciton ($\mathbf{X}_{\boldsymbol{0}}$) | -4.0 |
| Dark exciton ($\mathbf{X}_{\mathbf{D}}$) | -8.0 |
| Trion-exciton complexes ($\mathbf{XX}^{\mathbf{-}}$) | -4.0 |

**Supplementary Note 4. Excitation power and temperature dependence of PL spectra**

The low temperature PL spectra of BN encapsulated monolayer WSe_2_ as a function of excitation power (CW laser centered at 1.879 eV) is shown in Supplementary Fig. 2b.

We also measured the temperature dependent PL as shown in the Supplementary Fig. 2c and 2d, which indicates that all the excitonic states show a red shift with the increase of temperature. The PL intensities of the excitonic states all decrease as the temperature increase, except for the exciton state. The dark exciton phonon replica can survive up to the temperature of ~ 70 K.

#
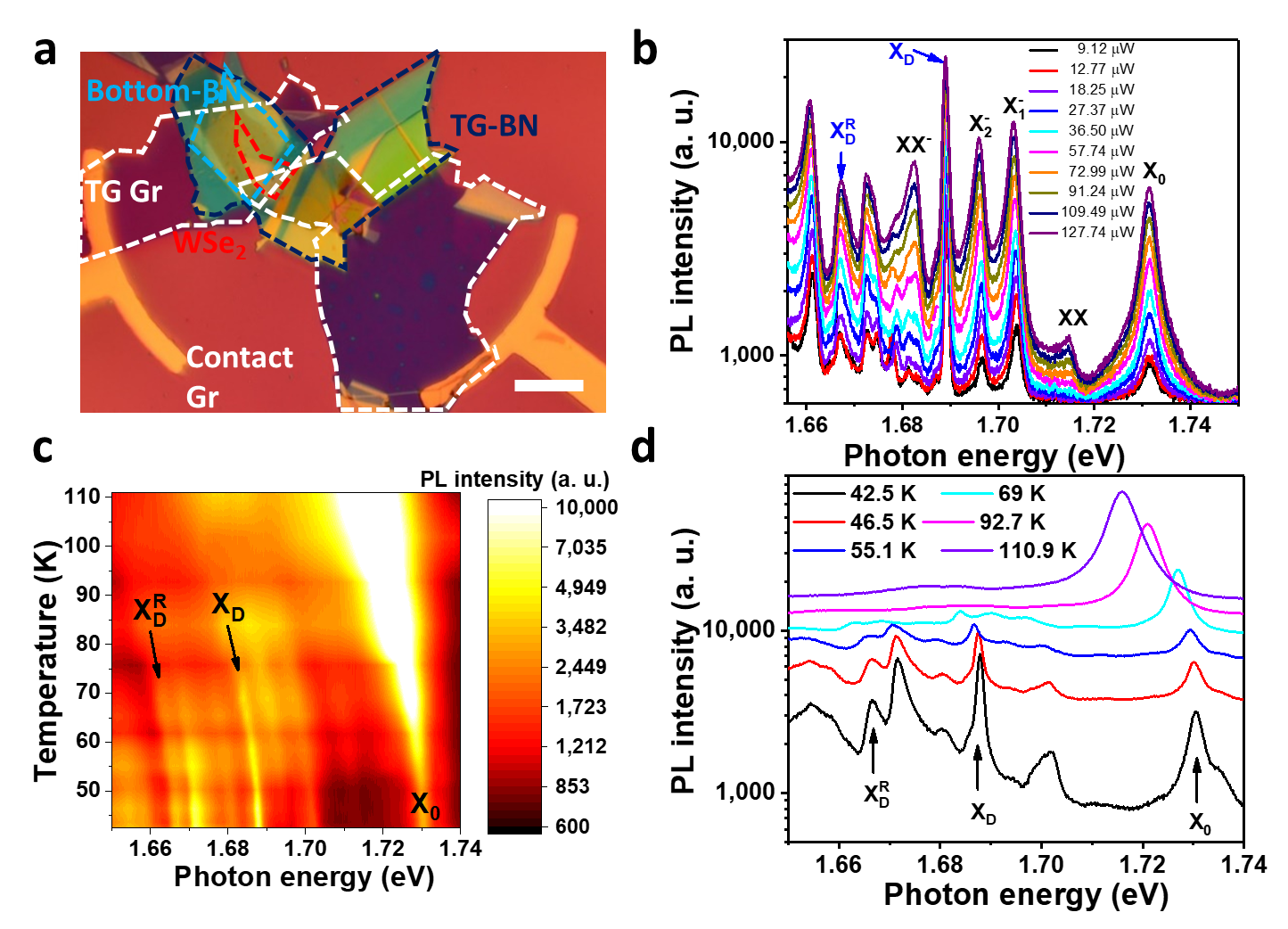


**Supplementary Figure 2. PL spectra of the monolayer WSe_2_.** (a) Optical microscope image of device 1, and the monolayer WSe_2_, top BN layer, bottom BN layer, contact graphene, and top gate graphene are outlined separately. Scale bar: 20 µm. (b) Low temperature (4.2 K) PL spectra of BN encapsulated monolayer WSe_2_ as a function of the excitation power. The excitation is a CW laser centered at 1.879 eV. (c) Color plot of the PL spectra as a function of the temperature, the color represents the PL intensity. The excitation is a CW laser centered at 1.797 eV. (d) Line-cuts of PL spectra at specific temperatures for c.

**Supplementary Note 5. The energy differences between** $\mathbf{X}_{\mathbf{D}}$ **and** $\mathbf{X}_{\mathbf{D}}^{\mathbf{R}}$ **for four devices**

The energy differences (Δ) between $X_{D}$ and $X_{D}^{R}$ for the different BN encapsulated monolayer WSe_2_ devices are shown in Supplementary Fig. 3. The first device (Supplementary Fig. 3a) is what we show in the main text and has the well-defined line shape with the narrowest linewidth. The corresponding Δ is $21.6 \pm0.1$ meV, in which the uncertainty is mostly determined by the grating we used (1200 g/mm). For the second device (Supplementary Fig. 3b) which we show gate dependence in the main text, the Δ is $21.3\pm0.2$ meV. The increased uncertainty is due to the grating we used (600 g/mm instead of 1200 g/mm). The Δ is 22.0 meV and 21.1 meV for device 3 and 4, respectively. Although the uncertainty due to the grating is about 0.2 meV, the experimental uncertainty mainly arises from the spectra quality for device 3 and 4. Although the energy differences from the four devices are consistent, we choose the value from the first device (21.6 meV) to present in the main text simply because it is the value with the least uncertainty.


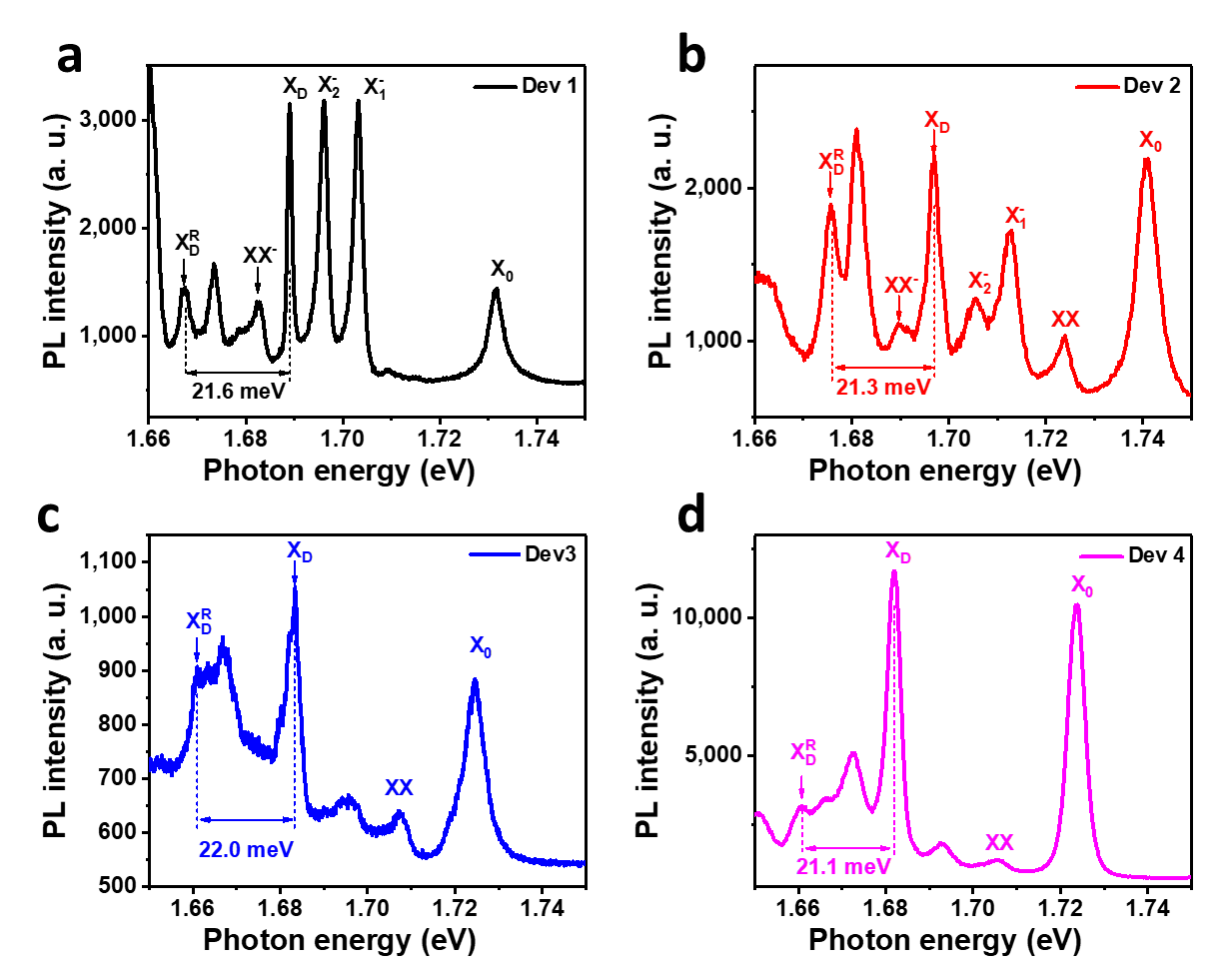


**Supplementary Figure 3. PL spectra for different devices. (a-d)** The energy differences between $X_{D}$ and $X_{D}^{R}$ for the devices 1-4 are 21.6 meV, 21.3 meV, 22.0 meV and 21.1 meV, respectively.

**Supplementary Note 6. Circular polarization of the PL from the dark-exciton phonon replica**

We performed the valley-resolved PL spectra measurement with the absence of the magnetic field in our lab (Supplementary Fig. 4a). We found that the dark exciton replica is also valley polarized with a valley polarization of ~ 16.7%, and the bright exciton possesses a valley polarization of ~ 39.5%. In contrast, the dark exciton is not valley-polarized.

Although the PL peak of both the dark exciton and dark-exciton phonon replica split into two peaks in the presence of out-of-plane magnetic field in the valley-resolved spectra, the behaviors of the dark exciton PL and the dark-exciton phonon replica are different. For the dark exciton, the PL intensity ratio for the blue-shifted high-energy peak to the red-shifted low-energy peak is about 1, as shown in Supplementary Fig. 4b (red dots). This is consistent with the fact that the dark exciton PL originates from the radiation from an out-of-plane dipole, which results in the equal intensities of the left circularized and right circular polarized components of the PL. However, the HE to the LE intensity ratio of the splitting PL peaks from dark-exciton phonon replica is much larger than 1 (blue dots in Supplementary Fig. 4b), suggesting the PL is circular polarized. This preferred circular polarization is consistent with our microscopic model which indicates that the PL of the dark exction phonon replica arises from the bright exciton emission.

**
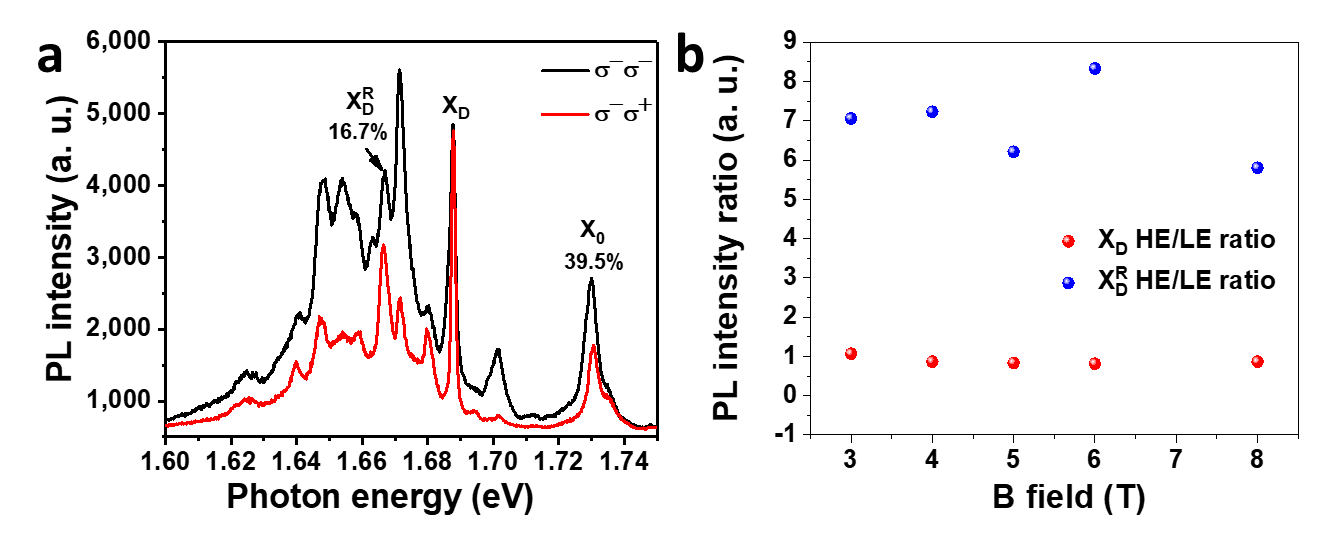
**

**Supplementary Figure 4. Valley-resolved PL spectra of monolayer WSe_2._** (a) Circular polarized PL spectra of monolayer WSe_2_ at 42 K, with no magnetic field applied. (b) The peak intensity ratio of the magnetic field splitting high-energy peak (HE) and low-energy peak (LE) for $X_{D}$ and $X_{D}^{R}$ as a function of the out-of-plane magnetic field.

**Supplementary Note 7. Valley-resolved magneto-PL spectra of Device 1 and Device 2**

**
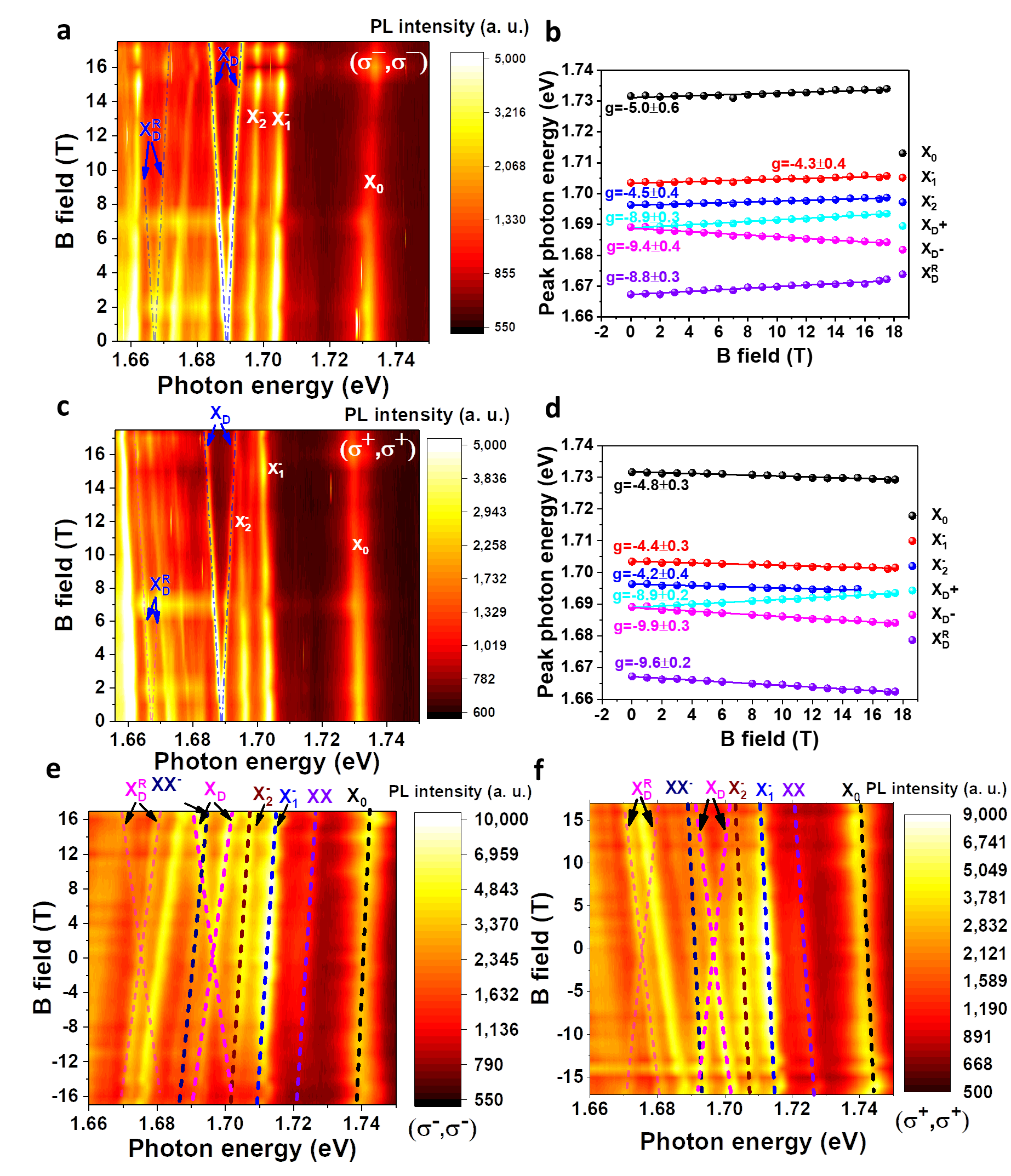
**

**Supplementary Figure 5. Valley-resolved magneto-PL spectra for device 1 and 2.** (a) Color plot of valley-resolved PL spectra as a function of the out-of-plane B field at 4.2 K for device 1 in the $(\sigma^{-},\sigma^{-})$ configuration, with the CW laser excitation centered at 1.879 eV and excitation power of 60 µW. (b) *g*-factors for different PL peaks of device 1 calculated from the Zeeman shift obtained from (a). (c) Color plot of valley-resolved PL spectra as a function of the out-of-plane B field at 4.2 K for device 1 in the $(\sigma^{+},\sigma^{+})$ configuration, with the CW laser excitation centered at 1.879 eV and excitation power of 60 µW. (d) *g*-factors for different PL peaks of device 1 calculated from the Zeeman shift obtained from (c). (e, f) Color plot of valley-resolved magneto PL spectra of device 2 for the ${(\sigma}^{-}{,\sigma}^{-})$ and $(\sigma^{+},\sigma^{+})$ configurations at 4.2 K, respectively. The spectra were taken with the CW laser excitation centered at 1.959 eV and the excitation power of 40 µW. The color represents the PL intensity.

At low temperature (4.2 K), the absorption of optical phonon is negligible due to small optical phonon population, and the exciton-phonon coupling will not modify the g factor of the dark exciton. On the other hand, the exciton-phonon coupling may weakly modify the g-factor of the dark-exciton phonon replica through modifying phonon frequency. However, we expect such change is small, comparable to the error bar of our measurement. Here we show the valley–resolved PL spectra of device 1 in the $(\sigma^{-},\sigma^{-})$ and $(\sigma^{+},\sigma^{+})$ configurations in the Supplementary Fig. 5a-d. Supplementary Fig. 5a and 5c shows the PL color plot as a function of the emission photon energy and the out-of-plane B field at 4.2 K for $(\sigma^{-},\sigma^{-})$ and $(\sigma^{+},\sigma^{+})$, respectively, with the excitation of a CW laser centered at 1.879 eV and excitation power of 60 µW. Different from the valley polarized spectra in the $(\sigma^{-},\sigma^{-})$ configuration, all the bright excitonic complexes PL peaks exhibit linear red shift instead of the blue shift. The PL peaks of the dark exciton and the replica, however, exhibit similar splitting behavior with the B field applied. It is interesting to note that the B field splitted low-energy (LE) peak is stronger in PL intensity than the high-energy (HE) peak for the dark-exciton phonon replica, consistent with our circular polarization discussion in the supplementary note 6.

The spectra g-factor in the main text is calculated by a linear fit of the Zeeman splitting between the $E^{K}$ and $E^{K^{'}}$. Meanwhile, the spectral *g*-factor can also be obtained through the fitting of the Zeeman shift (${\Delta E^{K^{'}}}=-\frac{1}{2}\mu_{B}gB$ and $\Delta E^{K}=\frac{1}{2}\mu_{B}gB$) as a function of B field for the light emission from either valley, and the values for different excitonic complexes are shown in Supplementary Fig. 5b and 5d. It is worth nothing that, since both valleys of the dark exciton are visible in either $(\sigma^{-},\sigma^{-})$ or $(\sigma^{+},\sigma^{+})$ configuration, the g-factor of the dark exciton can be extracted by either valley and we show both values in Fig. S5b and Fig. S5d. The values shown in Fig. S5b and S5d are consistent with what we present in the Fig. 2c of the main text, within the fitting uncertainty and the experimental uncertainty.

We also show the valley-resolved magneto PL spectra data of the device 2 for both the ${(\sigma}^{-},\sigma^{-})$ and $(\sigma^{+},\sigma^{+})$ configurations in supplementary Figs. 5e and 5f. We have shown the gate dependence data of the device 2 in the Fig. 3 of the main text. The B field dependence of the valley-resolved spectra for device 2 is consistent with our observation in device 1.

**Supplementary Note 8. Gate-voltage dependent PL spectra of three other WSe_2_ devices**

We have shown the gate voltage dependence of the PL spectra of device 2 in Fig. 3 of the main text. Here we also performed the gate-voltage dependent PL for the other three monolayer WSe_2_ devices. The gate dependence of the device 1 (we used the magnetic field dependence data from device 1 in Fig. 1 and Fig. 2 of the main text), is plotted in Supplementary Fig. 6a-b. We also show the data for device 3 and 4 in Supplementary Fig. 6c-d and Supplementary Fig. 6e-f, respectively. The gate-dependent PL for device 2 is presented in Fig. 3 of the main text and now shown here. The data for all the three devices shown here in Supplementary Fig. 6 demonstrate that the gate dependence of the replica PL closely mimics that of the dark exciton, consistent with the Fig. 3 of the main text (device 2), confirming the phonon replica picture.


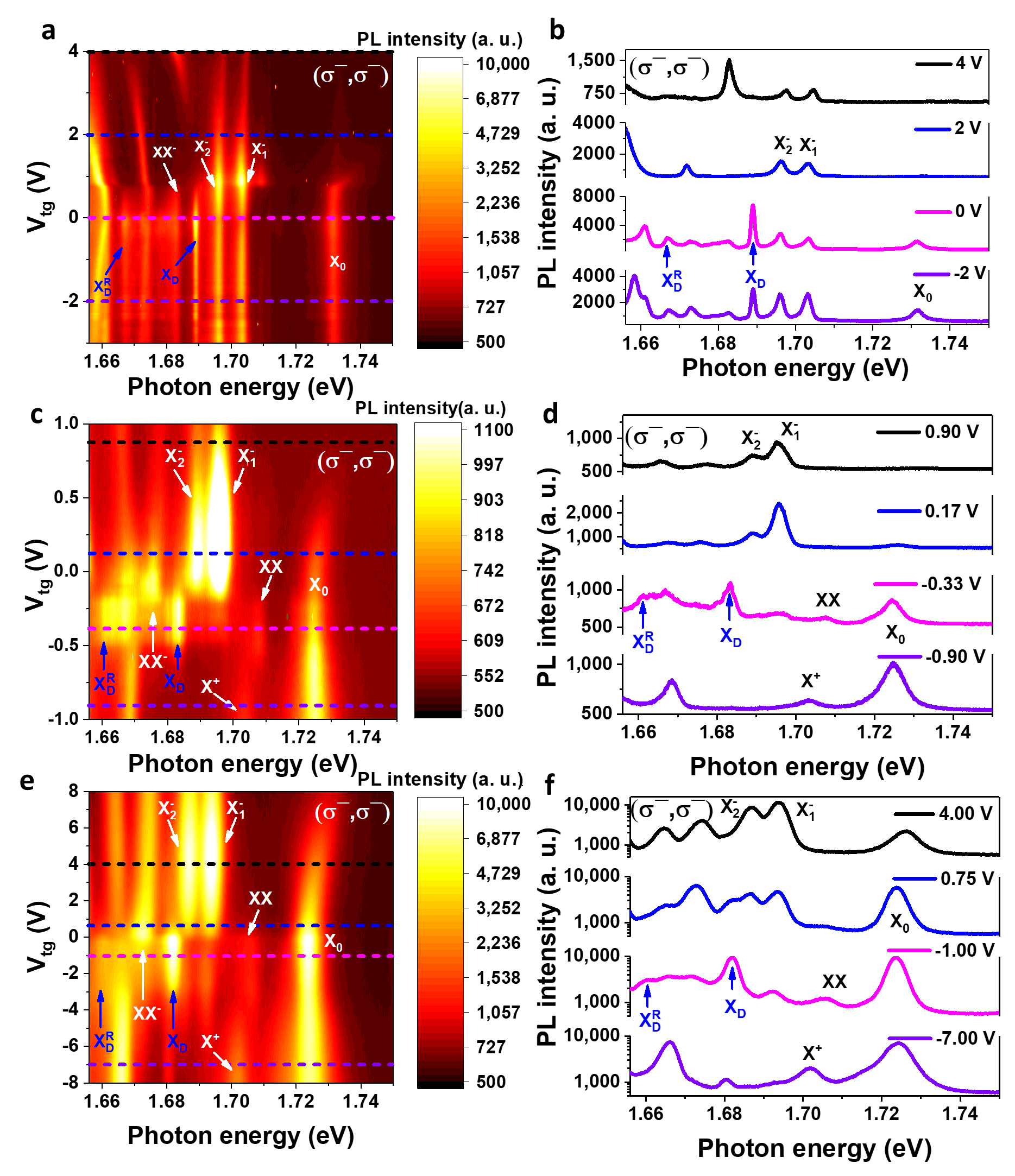


**Supplementary Figure 6. PL spectra of three other devices as a function of gate voltage.** (a, c, e) Color plot of the PL spectra as a function of the top gate voltage for device 1, device 3 and device 4, respectively. The color represents the PL intensity. (b, d, f) PL spectra at specific top gate voltages for the corresponding color plot (a, c, e).

**Supplementary Note 9. Time-resolved PL spectra for the dark exciton.**


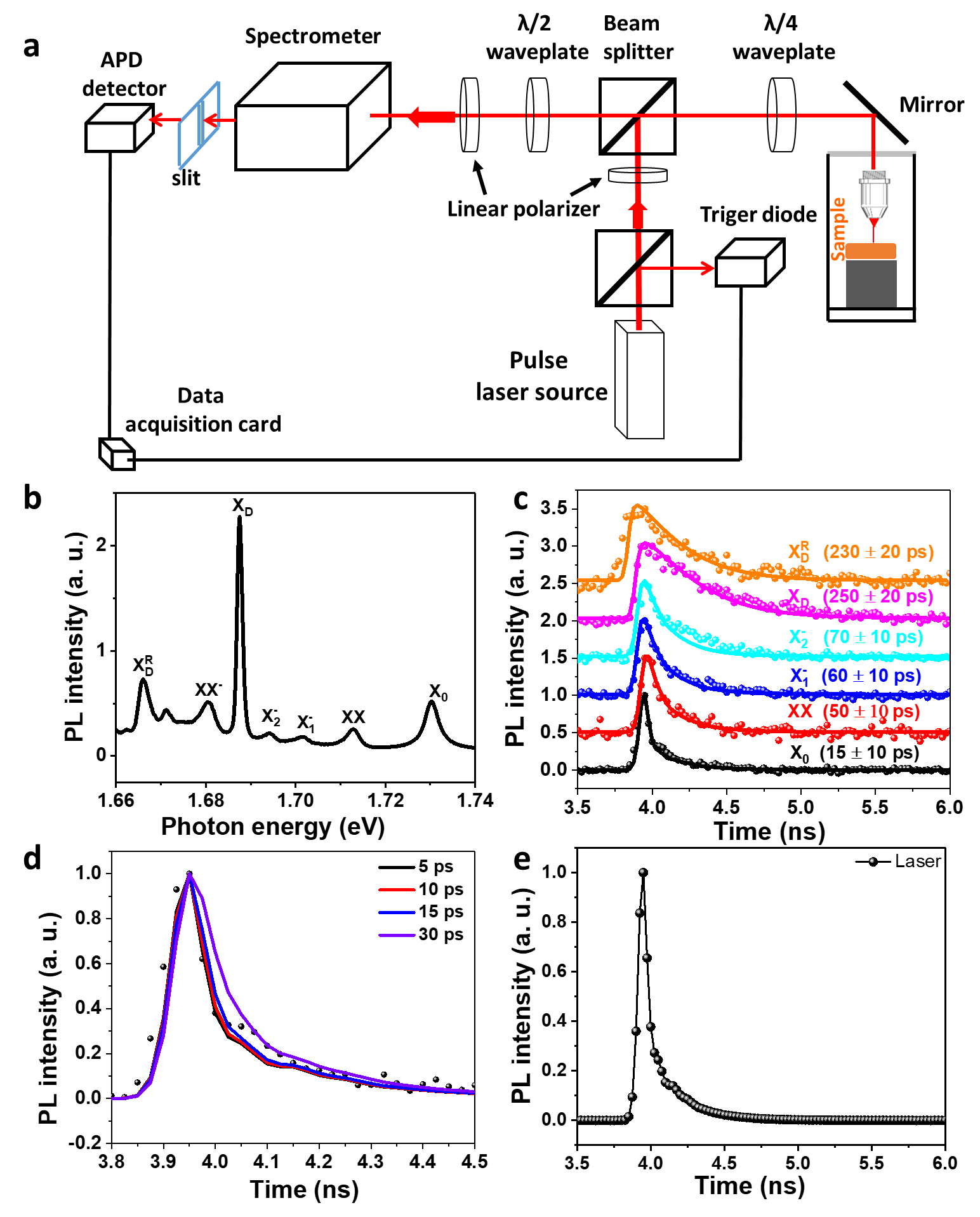


**Supplementary Figure 7. Time-resolved PL for different excitonic complexes.** (a) Schematic of the time-resolved PL measurement setup. (b) PL spectrum of the device 1 (we used data from device 1 in Fig. 1 and Fig. 2 in the main text) as a function of the emission photon energy at 42 K, with the pulse laser excitation centered at 1.959 eV with the excitation power of 30 µW. Under this excitation condition, the PL from exciton complexes such as the bright exciton ($X_{0}$), biexciton ($\mathrm{XX}$), negative trion 1 ($X_{1}^{-}$), negative trion 2 ($X_{2}^{-}$), dark exciton ($X_{0}$), and the charged biexciton ($\mathrm{XX}^{-}$) are all well resolved. (c) Time-resolved PL spectra (dots) for $X_{0}$, $X_{1}^{-}$, $X_{2}^{-}$ and $X_{D}$. The time-resolved PL data for different excitonic complexes are fitted ((solid line) by the single exponential function $I=Ae^{-t/\tau}$ convolved with the response of the laser as a kernel. The lifetime of $X_{D}$ and $X_{D}^{R}$ are 250$\pm$ 20 ps and 230 $\pm$ 20 ps, respectively, significantly longer than that of $X_{0}$ (15 ps). (d) Comparison of the different convolutions for $X_{0}$; (e) The response of excitation laser that is used as a kernel for the convolution.

The capability of resolving dark exciton allows us to probe the dynamics of dark exciton through time-resolved PL measurements directly. For device 1 (B field dependence data are shown in Fig. 1 and Fig. 2), we employ a pulse laser excitation with photon energy 1.959 eV and excitation power 30 µW to measure the PL spectra at 42 K without applying the top gate voltage, and the data is shown in Supplementary Fig. 7b. At this excitation condition, bright exciton ($X_{0}$), biexciton ($\mathrm{XX}$), trion1 ($X_{1}^{-}$), trion 2 ($X_{2}^{-}$), dark exciton ($X_{D}$) and the charged biexciton ($\mathrm{XX}^{-}$) are all clearly resolved.

We then select an emission photon energy with an uncertainty of $\pm1 meV$ to measure the time-resolved PL using the time-correlated single photon counting (TCSPC) technique, with the pulsed laser excitation centered at 1.959 eV and the excitation power of 30 µW (the pulsed laser is selected from a Fianium supercontinuum white laser source, 40 MHz covering the 410~2400 nm). One tunable slit is inserted between the monochrometer and the avalanche photodetector at the focal point of the monochrometer. The spectrum window we used for the collection of TRPL is around 0.6 nm with the excitation laser centered at 633 nm (1.959 eV), which was calibrated by using the laser line. The well-separated PL peaks allow us to record the time-resolved PL for bright exciton ($X_{0}$), negative trion1 ($X_{1}^{-}$), trion 2 ($X_{2}^{-}$), dark exciton ($X_{D}$) in supplementary Fig. 7c (solid dots). Taking into account the excitation laser response (Supplementary Fig. 7e), we perform convolution (solid lines in supplementary Fig. 7c) and extracted the lifetime of different excitonic complexes. The extracted lifetime of $X_{D}$ and $X_{D}^{R}$ are 250 $\pm$ 20 ps and 230 $\pm$ 20 ps, respectively, more than one order of magnitude longer than that of $X_{0}$ (~ 15 ps). The lifetime of the dark exciton phonon replica is essentially the same as that of the dark exciton within the measurement uncertainty, consistent with our interpretation. The long lifetime of the dark exciton allows us to use a quasi-equilibrium picture to estimate the phonon and dark exciton coupling. We also compare the different convolutions of the lifetime of the exciton, 5, 10, 15 and 30 ps, respectively (Supplementary Fig. 7d), which we can extract that the lifetime resolution is around 10 ps. The lifetimes of biexciton and trions also have an uncertainty of 10 ps while the dark exciton and dark exciton replica have an uncertainty of 20 ps.

**Theory calculation**

**Supplementary Note 10.** **Perturbation Theory Formulism of Phonon-Photon Emission from the Dark-Exciton Replica**

To quantitatively characterize the experimentally measured photoluminescence of the dark-exciton phonon replica, we write the Hamiltonian of the system, $H_{tot}$, as,

| $H_{tot}=H_{ex}+H_{ph}+H_{ex-ph}+H_{ex-l},$ | (4) |
| --- | --- |

where $H_{ex}$, $H_{ph}$, $H_{ex-ph}$, and $H_{ex-l}$ correspond to the Hamiltonian of exciton, phonon, exciton-phonon coupling, exciton-light coupling, respectively. Here, the exciton degree of freedom refers to those in the K valley. The perturbing Hamiltonians are the two coupling terms $H_{ex-ph}$ and $H_{ex-l}$. The photons are treated as classical electro-magnetic fields in this approach.

For the exciton Hamiltonian $H_{ex}$, we consider a basis set consisting of 1*s* dark excitons the 1*s* bright excitons.

| $H_{ex}=\Sigma_{\boldsymbol{Q}}\left[ E_{D}\left( \boldsymbol{Q} \right)\hat{c}_{D}^{\dagger}\left( \boldsymbol{Q} \right)\hat{c}_{D}\left( \boldsymbol{Q} \right)+E_{0}\left( \boldsymbol{Q} \right)\hat{c}_{0}^{\dagger}\left( \boldsymbol{Q} \right)\hat{c}_{0}\left( \boldsymbol{Q} \right) \right],$ | (5) |
| --- | --- |

where $\hat{c}\left( \boldsymbol{Q} \right)$ and $\hat{c}^{\dagger}\left( \boldsymbol{Q} \right)$ are annihilation and creation operator of excitons with momentum $\boldsymbol{Q}$, respectively. $E_{D}\left( \boldsymbol{Q} \right)$ and $E_{0}\left( \boldsymbol{Q} \right)$ are the dark and bright 1s exciton energies, respectively, in the K valley with a momentum $\boldsymbol{Q}$**.** Higher-energy exciton states, such as 2*p* and 2*s*, are not included, because the energy splitting between the 1*s* dark exciton and these excitons (≥ 200 meV) are much larger than the splitting between the 1*s* dark and bright excitons (40-50 meV). Their coupling to the 1*s* dark excitons due to the exciton-phonon coupling is hence neglected in this work.

For the phonon Hamiltonian $H_{ph}$, we consider the two-fold degenerate $E''$ phonon mode identified in our experiment,

| $H_{ph}=\Sigma_{n\boldsymbol{q}}\hbar\omega_{n}\left( \boldsymbol{q} \right)\left[ \hat{b}_{n}^{\dagger}\left( \boldsymbol{q} \right)\hat{b}_{n}\left( \boldsymbol{q} \right)+\frac{1}{2} \right],$ | (6) |
| --- | --- |

where $\omega_{n}\left( \boldsymbol{q} \right)$ is the frequency of the $E''$ phonon mode $n$ (1 or 2) at wavevector $\boldsymbol{q}$. The operators $\hat{b}_{n}^{\dagger}\left( \boldsymbol{q} \right)$ and $\hat{b}_{n}\left( \boldsymbol{q} \right)$ are the creation and annihilation operators of the $E''$ phonon mode $n$ (1 or 2) at wavevector $\boldsymbol{q}$, respectively. We ignore the anharmonic effects of $E''$ phonons.

The exciton-phonon coupling $H_{ex-ph}$ describe the coupling between the bright and dark excitons by an $E''$ phonon mode, having a form,

| $H_{ex-ph}=\Sigma_{\boldsymbol{q},\boldsymbol{Q},n}g_{0Dn}^{ex}\left( \boldsymbol{Q,q} \right)\hat{c}_{0}^{\dagger}\left( \boldsymbol{Q}+\boldsymbol{q} \right)\hat{c}_{D}\left( \boldsymbol{Q} \right)\left[ \hat{b}_{n}\left( \boldsymbol{q} \right)+\hat{b}_{n}^{\dagger}(-\boldsymbol{q}) \right]+\Sigma_{\boldsymbol{q},\boldsymbol{Q},n}g_{D0n}^{ex}\left( \boldsymbol{Q,q} \right)\hat{c}_{D}^{\dagger}\left( \boldsymbol{Q}+\boldsymbol{q} \right)\hat{c}_{0}\left( \boldsymbol{Q} \right)\left[ \hat{b}_{n}\left( \boldsymbol{q} \right)+\hat{b}_{n}^{\dagger}(-\boldsymbol{q}) \right],$ | (7) |
| --- | --- |

where $g_{0Dn}^{ex}\left( \boldsymbol{Q,q} \right)$ is the inter-exciton-band exciton-phonon matrix element. Intra-exciton-band exciton-phonon coupling that does not contribute to the photoluminescence of dark exciton phonon replica are not included. Higher order couplings that involve two-phonon terms are not included.

For the exciton photon emission process, we consider light with electric field polarized in-plane. For this polarization condition, only bright excitons with momentum nearly at 0 couple to light directly. Here, we consider light emitted along the out-of-plane direction. By momentum conservation, such light only couples to the $\boldsymbol{Q}=\boldsymbol{0}$ bright excitons.

| $H_{ex-ph}=M_{1s}\hat{a}^{\dagger}\hat{c}_{0}\left( \boldsymbol{0} \right)+h.c.,$ | (8) |
| --- | --- |

where $M_{1s}$ is the transition dipole of the 1*s* bright exciton. $\hat{a}^{\dagger}$ is the photon creation operator. The polarization (linear or circular) dependence is implicitly included in $M_{1s}$ and $\hat{a}$.

In the second-order perturbation theory, the transition rate of simultaneous emitting an $E''$ phonon and a photon from the dark exciton with momentum $\boldsymbol{Q}$ is^9^,

| $W\left( \omega_{D}^{R} \right)={\Sigma_{n}\left\vert\frac{\left\langle\Phi_{D}\left( \boldsymbol{Q} \right),0\left\vert H_{ex-ph} \right\vert\Phi_{0}\left( \boldsymbol{0} \right),1_{n,\boldsymbol{Q}} \right\rangle\left\langle\Phi_{0}\left( \boldsymbol{0} \right),1_{n,\boldsymbol{Q}}\left\vert H_{ex-l} \right\vert\Phi_{G},1_{n,\boldsymbol{Q}} \right\rangle}{E_{D}\left( \boldsymbol{Q} \right)-E_{0}\left( \boldsymbol{0} \right)-\hbar\omega_{n}\left( \boldsymbol{Q} \right)} \right\vert}^{2}\delta\left( E_{D}\left( \boldsymbol{Q} \right)-h\omega_{n}\left( \boldsymbol{Q} \right)-\hbar\omega_{D}^{R} \right)$ | (9) |
| --- | --- |

Where $\Phi_{D}$, $\Phi_{0}$, and $\Phi_{G}$ are the wavefunctions of the dark exciton, bright exciton, and ground state, respectively. The numbers, 0 and 1, label the number of $E''$ phonon involved in each process.

At the experimental temperature of 4.2 K, the thermal energies of dark excitons are in the order of 0.36 meV. With an effective mass of dark excitons at 1.1 $M_{0}$ (electron rest mass), the momentum corresponding to this thermal energy is 0.1 Å^-1^ (< 5 % reciprocal lattice vector). Because of the dark excitons are all located at the band bottom, the exciton-phonon coupling strength between these dark excitons and the bright $\boldsymbol{Q}=\mathbf{0}$ exciton is essentially a constant. We therefore use the $\boldsymbol{Q=0}$ dark excitons to calculate the phonon-photon emission probability. Equation 9 then simplifies to,

| $W\left( \omega_{D}^{R} \right)\approx{\Sigma_{n}\left\vert\frac{\left\langle\Phi_{D}\left( \boldsymbol{Q} \right),0\left\vert H_{ex-ph} \right\vert\Phi_{0}\left( \boldsymbol{0} \right),1_{n,\boldsymbol{Q}} \right\rangle}{E_{D}-E_{0}-\hbar\omega_{E^{''}}} \right\vert}^{2}\left\vert M_{1s} \right\vert^{2} \delta\left( E_{D}-h\omega_{E^{''}}-\hbar\omega_{D}^{R} \right)$ | (10) |
| --- | --- |

In this expression, $\left| M_{1s} \right|^{2}$ is the module squared transition matrix element between the ground state and the 1*s* state. Under the displacement of one phonon, the transition matrix element is almost unchanged. For notational simplicity, we have also defined $E_{D}=E_{D}\left( \boldsymbol{Q} \right)$, $E_{0}=E_{0}\left( \boldsymbol{0} \right)$, and $\omega_{E^{''}}=\omega_{n}\left( \boldsymbol{0} \right)$**.** The two branches of the phonon modes (n=1, 2) are degenerated in energy at $Q=0$ (see Supplementary Note 13).

**Supplementary Note 11. First-principles Calculation of Electron-Phonon Coupling**

We use a frozen phonon method^10^ to calculate the coupling between the two spin-split conduction bands caused by the $E''$ phonon.

For the quantum description of the phonon, the relation between the phonon annihilation (creation) operator and the displacement of the Se atom in the unit cell $p$ along the $\alpha$ axis is^11^,

| $\Delta\tau_{Se,\alpha,p}=\sqrt{\frac{M_{0}}{N_{p}M_{Se}}} \Sigma_{n, \boldsymbol{q}}e^{i\boldsymbol{q}\cdot R_{p}}e_{Se,\alpha, n}\left( \boldsymbol{q} \right)l_{\boldsymbol{q},n}\left( \hat{b}_{n}\left( \boldsymbol{q} \right)+\hat{b}_{n}^{\dagger}(-\boldsymbol{q}) \right)$ | (11) |
| --- | --- |

where $l_{\boldsymbol{q},n}=\sqrt{\frac{\hbar}{2M_{0}\omega_{n}\left( \boldsymbol{q} \right)}}$.$M_{0}$ is the electron rest mass. $N_{p}$ is number of unit cells of the crystal. $\boldsymbol{R}_{p}$ is the coordinate of the unit cell $p$. $e_{Se,\alpha, n}\left( \boldsymbol{q} \right)$ is the phonon eigenvector (Se component). $M_{Se}$ is the mass of the Se atom. For the two degenerate $E''$ phonons ($n=1$ and $n=2$) of interest, we let the Se atoms in the $n=1$ mode vibrate along the x axis, and those in the $n=2$ mode vibrate along the y axis. Using this convention, the eigenvectors are $e_{Se1,x, 1}=\frac{1}{\sqrt{2}}$ and $e_{Se2,x, 1}=-\frac{1}{\sqrt{2}}$ (the index Se1 stands for Se in the top layer and Se2 stands for Se in the bottom layer). For the second $E''$ phonon, $e_{Se1,y,2}=\frac{1}{\sqrt{2}}$ and $e_{Se2,y,2}=-\frac{1}{\sqrt{2}}$. For both mode, the two Se atoms in one unit cell move in opposite directions.

The zero point displacement of the Se atom caused by the $E''$ phonon with a frequency $\omega_{E^{''}}=21.8 meV$ is,

| $l_{Se,E^{''}}=\sqrt{\frac{\hbar}{2M_{\mathrm{Se}}\omega_{E^{''}}}}=0.035 Å$ | (12) |
| --- | --- |

The phonon coupling matrix element between band states of the lower and upper conduction bands, $u_{c_{1},\boldsymbol{k}+\boldsymbol{q}}$ at $c_{1}$ and $u_{c_{2},\boldsymbol{k}}$ at $c_{2}$, have a general form,

| $g_{c_{1}c_{2}, n}^{e}\left( \boldsymbol{k,q} \right)=\left\langle u_{c_{1},\boldsymbol{k}+\boldsymbol{q}} \right\vert\Delta_{\boldsymbol{q}, n}V\left\vert u_{c_{2},\boldsymbol{k}} \right\rangle$ | (13) |
| --- | --- |

where $V$ is the self-consistent potential including the spin-orbit coupling term, and ***k*** is the wave vector of the electron . This matrix element goes into the electron-phonon Hamiltonian as

| $H_{e-ph}=\frac{1}{\sqrt{N_{p}}}\Sigma_{\boldsymbol{k,q,}n}g_{c_{1}c_{2}, n}^{e}\left( \boldsymbol{k,q} \right)\hat{c}_{c_{1}}^{e\dagger}\left( \boldsymbol{k}+\boldsymbol{q} \right)\hat{c}_{c_{2}}^{e}\left( \boldsymbol{k} \right)\left( \hat{b}_{n}\left( \boldsymbol{q} \right)+\hat{b}_{n}^{\dagger}(-\boldsymbol{q}) \right)+ h.c.$ | (14) |
| --- | --- |

$\hat{c}_{c_{1}}^{e\dagger}\left( \boldsymbol{k}+\boldsymbol{q} \right)$ and $\hat{c}_{c_{2}}^{e}\left( \boldsymbol{k} \right)$ are electron creation and annihilation operators, respectively.

Considering an $E''$ phonon mode $n$ at the Γ-point point, which has eigenvector $e_{Se,x, n}$, the change of the self-consistent potential in Equation (12) is normalized by a displacement $\tau$ of the Se atom,

| $\Delta V=\frac{1}{\sqrt{2}} l_{Se,E^{''}}\frac{V\left( \tau\right)-V(0)}{\tau}$ | (15) |
| --- | --- |

Here, we take $\tau=0.035 Å$, the same as the zero-point displacement amplitude. The displacement $\tau$ is taken along the x axis in our calculation and opposite between the two Se atoms in one unit cell. For this structure, we calculate the expectation value of $\hat{s}_{x}$ and $\hat{s}_{y}$ of $c_{1}$ and $c_{2}$ wavefunctions at the K point. For $c_{1}$, we obtain $s_{x}=0.27 \hbar$ and $s_{y}=0.$For $c_{2}$, we obtain $s_{x}=-0.27 \hbar$ and $s_{y}=0$. We plot the spins and band structure in the displacement in $n=1$ in supplementary Fig. 8. Since the conduction band spin splitting is 40 meV in the unperturbed structure, we deduce that the matrix element between $c_{1}$ and $c_{2}$ $\left\langle u_{c_{1},\boldsymbol{k}} \right|H\left( \tau\right)\left| u_{c_{2},\boldsymbol{k}} \right\rangle$ is ~ 13 meV.


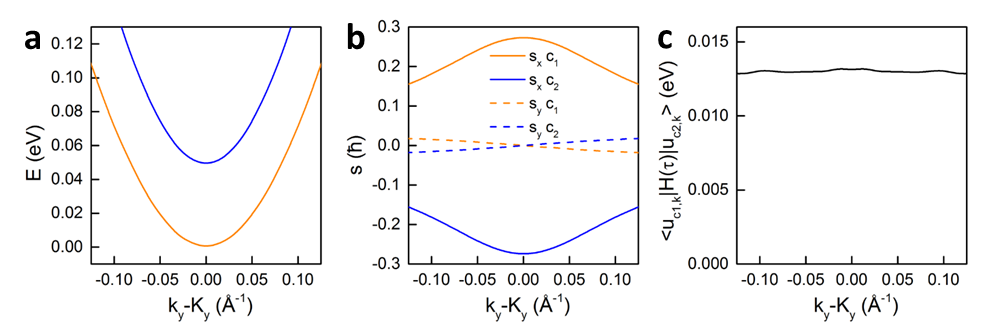


**Supplementary Figure 8. Electron-phonon coupling from frozen phonon calculations.** The k-space path is taken along the y direction, with k_x_ = K_x_. (a) Conduction band structures after displacement. The two bands have opposite spins at each k point. (b) $s_{x}$ and $s_{y}$ of the two conduction bands as a function of k. (c) the matrix element between $c_{1}$ and $c_{2}$ $\left\langle u_{c_{1},\boldsymbol{k}} \right|H\left( \tau\right)\left| u_{c_{2},\boldsymbol{k}} \right\rangle$ as a function of k.

Because $c_{1}$ and $c_{2}$ wavefunctions at K have opposite spins, the matrix element $\left\langle u_{c_{1},\boldsymbol{K}} \right|H^{KE}\left| u_{c_{2},\boldsymbol{K}} \right\rangle=0$, where $H^{KE}$ is the kinetic energy part of the Hamiltonian. We therefore have,

| $\left\langle u_{c_{1},\boldsymbol{K}} \right\vert V\left( \tau\right)-V\left( 0 \right)\left\vert u_{c_{2},\boldsymbol{K}} \right\rangle=\left\langle u_{c_{1},\boldsymbol{K}} \right\vert H\left( \tau\right)-H\left( 0 \right)\left\vert u_{c_{2},\boldsymbol{K}} \right\rangle=\left\langle u_{c_{1},\boldsymbol{K}} \right\vert H\left( \tau\right)\left\vert u_{c_{2},\boldsymbol{K}} \right\rangle$ | (16) |
| --- | --- |

where $H\left( \tau\right)$ is the total Hamiltonian when the Se atom is at a displacement of $\tau$, and $H\left( 0 \right)$ is the total Hamiltonian when the Se atom is at the original equilibrium position (no vibration).

Taking together Equation (12), (13), and (15), we have $g_{c_{1}c_{2}, E^{''}}^{e}\left( \boldsymbol{K,0} \right) \sim$9.1 meV.

We further calculate $g_{c_{1}c_{2}, E^{''}}^{e}\left( \boldsymbol{k,0} \right)$ in the vicinity of the K point. Our results show that $g_{c_{1}c_{2}, E^{''}}^{e}\left( \boldsymbol{k,0} \right)$ is nearly a constant in the area where exciton wavefunction lives (Supplementary Fig. 8c), i.e.,

| $g_{c_{1}c_{2}, E^{''}}^{e}\left( \boldsymbol{k,0} \right)\approx g_{c_{1}c_{2}, E^{''}}^{e}\left( \boldsymbol{K,0} \right)$ | (17) |
| --- | --- |

On the other hand, the change of the valence band spin is negligible ($s_{x}\left( s_{y} \right)<0.005 \hbar$ at K) under the same displacement $\tau$. So the $E''$ phonon coupling with the holes is negligible compared with its coupling with the electrons.

**Supplementary Note 12.** **Exciton-Phonon Coupling from Electron-Phonon Coupling**

In monolayer WSe_2_, the wavefunctions of bright and dark 1s excitons are respectively $\Phi_{0}\left( \boldsymbol{0} \right)\boldsymbol{=}\Sigma_{k}A_{k}^{0}\phi_{c2}\left( k \right)\phi_{v}^{*}\left( k \right)$ and $\Phi_{D}\left( \boldsymbol{0} \right)\boldsymbol{=}\Sigma_{k}A_{k}^{D}\phi_{c1}\left( k \right)\phi_{v}^{*}\left( k \right)$. Under the same gauge (making the exciton wavefunction $A_{k}^{0}$ real), we plot the $A_{k}^{0}$ and $A_{k}^{D}$ around the K point in the Brillouin zone in supplementary Fig. 9, calculated from the *ab intio* GW-BSE method^12,13^.


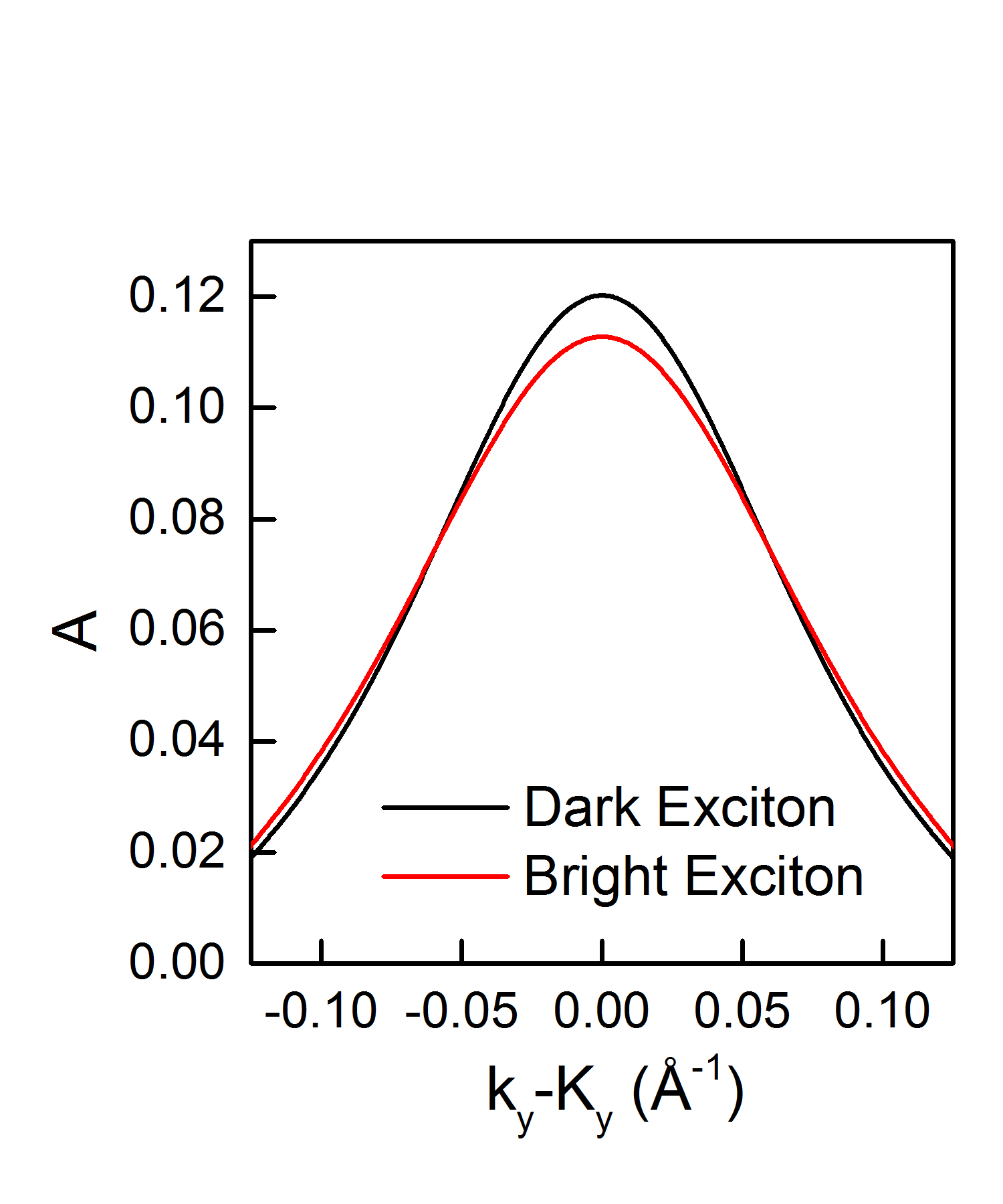


**Supplementary Figure 9. Dark and bright exciton wavefunctions in k-space.** The black and red curves correspond to dark exciton and bright excitons, respectively. The k-space path is taken along the y direction, with k_x_ = K_x_.

Because the bright and dark excitons have very similar k-space wavefunctions, we use

| $A_{k}^{0}\approx A_{k}^{D}$ | (18) |
| --- | --- |

in the calculations of exciton-phonon coupling. The k-space distribution of the exciton wavefunction shows that both excitons are localized in the vicinity of the K point^14^.

We now analyze the phonon coupling of the bright and dark excitons, which is the underlying physical mechanism of photoluminescence of the dark-exciton phonon replica. Under the GW-Bethe-Salpeter equation framework from the quantum many-body perturbation theory^12,13^, the exciton effective Hamiltonian $H_{ex}$ contains the quasi-electron Hamiltonian $H_{e}$ of the conduction band electrons, quasi-hole Hamiltonian $H_{h}$ of the valence band holes, and the electron-hole interaction $H_{e-h}$. For monolayer WSe_2_, We have analyzed in Supplementary Note 11 that $H_{e}$ and electron states could strongly interact with the $E''$ phonons via phonon-coupling $c_{1}$ and $c_{2}$, whereas $H_{h}$ and hole states are not affected by the $E''$ phonons. The electron-hole interaction $H_{e-h}$ is not affected by the $E''$ phonons in the first order, because the electron-hole interaction is spin-conserving, whereas the spin quantum numbers of the bright ($s_{z}=0$) and dark excitons ($s_{z}=\pm\hbar$) are different. For the purpose of studying the roles of $E''$ phonon in coupling the bright and dark excitons, we have,

| $\left\langle\Phi_{D}\left( \boldsymbol{0} \right),0\left\vert H_{ex-ph} \right\vert\Phi_{0}\left( \boldsymbol{0} \right),1_{n,\boldsymbol{0}} \right\rangle=\left\langle\Phi_{D}\left( \boldsymbol{0} \right),0\left\vert H_{e-ph} \right\vert\Phi_{0}\left( \boldsymbol{0} \right),1_{n,\boldsymbol{0}} \right\rangle$ | (19) |
| --- | --- |

Combining Equation (19) and Equation (14), the exciton-phonon coupling matrix elements between the two excitons have a simple form

| $\left\langle\Phi_{D}\left( \boldsymbol{0} \right),0\left\vert H_{ex-ph} \right\vert\Phi_{0}\left( \boldsymbol{0} \right),1_{n,\boldsymbol{0}} \right\rangle=\Sigma_{k}A_{k}^{D*}A_{k}^{0}g_{c_{1}c_{2}, E^{''}}^{e}\left( \boldsymbol{k,0} \right)$ | (20) |
| --- | --- |

We now further simply this expression by taking Equation (18) and Equation (17). Finally, we have

| $\left\langle\Phi_{D}\left( \boldsymbol{0} \right),0\left\vert H_{ex-ph} \right\vert\Phi_{0}\left( \boldsymbol{0} \right),1_{n,\boldsymbol{0}} \right\rangle=g_{c_{1}c_{2}, E^{''}}^{e}\left( \boldsymbol{K,0} \right)=9.1 \mathrm{meV}$ | (21) |
| --- | --- |

Since the $E''$ phonons at the Γ point are doubly degenerate, we further define the two phonon modes in a chiral basis^15^. In this new basis, the $n=1$ mode has $e_{Se1,x, 1}=\frac{1}{2}$ and $e_{Se1,y,2}=\frac{i}{2}$ for Se1, and $e_{Se2,x, 1}=-\frac{1}{2}$ and $e_{Se2,y,2}=-\frac{i}{2}$ for Se2. In $n=2$ mode, $e_{Se1,x, 1}=\frac{1}{2}$ and $e_{Se1,y,2}=-\frac{i}{2}$ for Se1, and $e_{Se2,x, 1}=-\frac{1}{2}$ and $e_{Se2,y,2}=\frac{i}{2}$ for Se2. In this new basis, the angular momentum of the two doubly degenerate modes are +1 and -1, respectively. Due to the angular momentum conservation, the n=1 mode is allowed to couple the dark to the bright exciton, whereas such coupling due to the n=2 mode is forbidden. Equation (9) can then be written into a simpler form,

| $W\left( \omega_{D}^{R} \right)\approx\left\vert\frac{\left\langle\Phi_{D}\left( \boldsymbol{0} \right)\left\vert H_{ex-ph} \right\vert\Phi_{0}\left( \boldsymbol{0} \right) \right\rangle}{E_{D}-E_{0}-\hbar\omega_{E^{''}}} \right\vert^{2}\left\vert M_{1s} \right\vert^{2} \delta\left( E_{D}-h\omega_{E^{''}}-\hbar\omega_{D}^{R} \right)$ | (22) |
| --- | --- |

where the n=1 mode is solely responsible for the exciton-phonon coupling at the K valley.

**Supplementary Note 13. First-principles calculation of phonon modes in monolayer WSe_2_**

The linear response approach^16–18^ base on density functional theory (DFT), which is implemented in the Quantum Espresso package^19^, is applied to study lattice vibrations and phonons of monolayer WSe_2_. The frozen phonon calculations^10^ of electron-phonon coupling is based on density functional theory as implemented in the Quantum Espresso package. The plane-wave energy cutoff is set to be 90 Ry using norm-conserving pseudopotentials with semi-core electrons included. In the DFPT calculations, we employ a 6×6×1 k-grid sampling in the reciprocal space for the converged phonon dispersion presented in Supplementary Figure 10. As seen from Supplementary Figure 10a, there are two degenerated optical phonon models with a vibration energy of 21.8 meV, in excellent agreement with our observation of 21.6 meV. The lattice vibration of these two modes is schematically plotted in the lower panel of Supplementary Figure 10b and 10c. These in-plane optical vibrational mode will keep the momentum conservation during optical transitions.


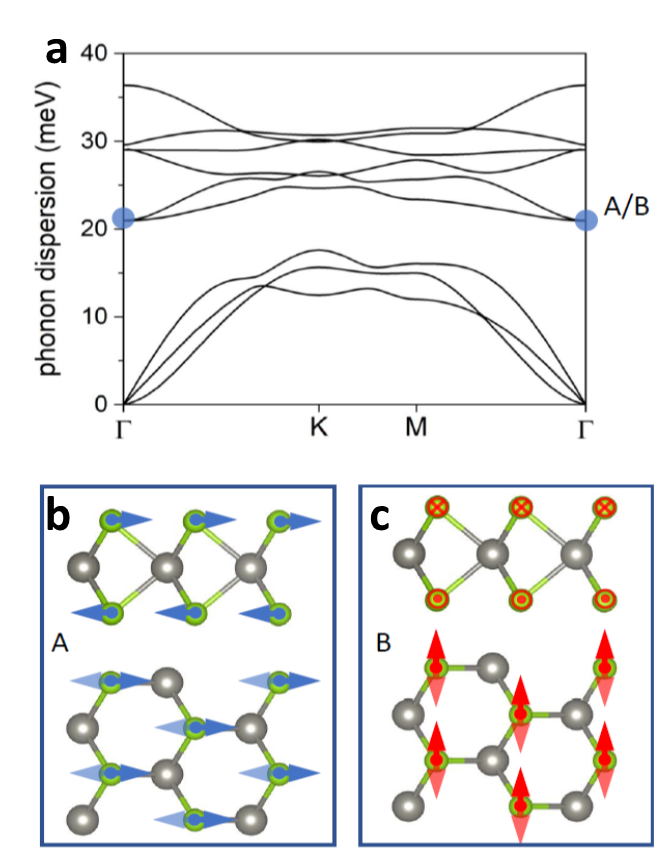


**Supplementary Figure 10. DFT calculation of the phonons in monolayer WSe_2_.** (a) The phonon dispersion of suspended monolayer WSe_2_. Our interested optical phonons (A/B) are degenerated at the Γ point, with an energy of 21.6 meV. (b, c) The dies and top views of corresponding lattice vibrations are plotted respectively.

**Supplementary References**

1. Li, Z. *et al.* Revealing the Biexciton and Trion-exciton Complexes in BN Encapsulated WSe_2_. *Nat. Commun.* **9,** 3719 (2018).

2. Wang, L. *et al.* One-Dimensional Electrical Contact to a Two-Dimensional Material. *Science.* **342,** 614–617 (2013).

3. Macneill, D. *et al.* Breaking of valley degeneracy by magnetic field in monolayer MoSe_2_. *Phys. Rev. Lett.* **114,** 037401 (2015).

4. Srivastava, A. *et al.* Valley Zeeman effect in elementary optical excitations of monolayer WSe_2_. *Nat. Phys.* **11,** 141–147 (2015).

5. Aivazian, G. *et al.* Magnetic control of valley pseudospin in monolayer WSe_2_. *Nat. Phys.* **11,** 148–152 (2015).

6. Nagler, P. *et al.* Giant Zeeman splitting inducing near-unity valley polarization in van der Waals heterostructures. *Nat. Commun.* **8,** 1551 (2017).

7. Stier, A. V., McCreary, K. M., Jonker, B. T., Kono, J. & Crooker, S. A. Exciton diamagnetic shifts and valley Zeeman effects in monolayer WS_2_ and MoS_2_ to 65 Tesla. *Nat. Commun.* **7,** 10643 (2016).

8. You, Y. *et al.* Observation of biexcitons in monolayer WSe_2_. *Nat. Phys.* **11,** 477–481 (2015).

9. Toyozawa, Y. Interband effect of lattice vibrations in the exciton absorption spectra. *J. Phys. Chem. Solids* **25,** 59–71 (1964).

10. Dacorogna, M. M., Cohen, M. L. & Lam, P. K. Self-Consistent Calculation of the q Dependence of the Electron-Phonon Coupling in Aluminum. *Phys. Rev. Lett.* **55,** 837–840 (1985).

11. Giustino, F. Electron-phonon interactions from first principles. *Rev. Mod. Phys.* **89,** 015003 (2017).

12. Rohlfing, M. & Louie, S. G. Electron-hole excitations and optical spectra from first principles. *Phys. Rev. B - Condens. Matter Mater. Phys.* **62,** 4927–4944 (2000).

13. Hybertsen, M. S. & Louie, S. G. Electron correlation in semiconductors and insulators: Band gaps and quasiparticle energies. *Phys. Rev. B* **34,** 5390–5413 (1986).

14. Zhang, X. X. *et al.* Magnetic brightening and control of dark excitons in monolayer WSe_2_. *Nat. Nanotechnol.* **12,** 883–888 (2017).

15. Zhang, L. & Niu, Q. Chiral Phonons at High-Symmetry Points in Monolayer Hexagonal Lattices. *Phys. Rev. Lett.* **115,** 115502 (2015).

16. Baroni, S. & Resta, R. Ab initio calculation of the low-frequency Raman cross section in silicon. *Phys. Rev. B* **33,** 5969–5971 (1986).

17. Xavier Gonze, Douglas C. Allan, and M. P. T. Dielectric tensor, effective charges, and phonons in α-quartz by variational density-functional perturbation theory. *Phys. Rev. Lett.* **68,** 3603–3606 (1992).

18. Baroni, S., Giannozzi, P. & Testa, A. Greens-function approach to linear response in solids. *Phys. Rev. Lett.* **58,** 1861–1864 (1987).

19. Wentzcovitch, P. G. *et al.* QUANTUM ESPRESSO: A modular and open-source software project for quantum simulations of materials. *J. Phys. Condens. Matter* **21,** 395502 (2009).
